# MicroRNA-95-3p serves as a contributor to cisplatin resistance in human gastric cancer cells by targeting EMP1/PI3K/AKT signaling

**DOI:** 10.18632/aging.202679

**Published:** 2021-03-10

**Authors:** Qingfeng Ni, Yan Zhang, Ran Tao, Xiaolong Li, Jianwei Zhu

**Affiliations:** 1 Department of General Surgery, Affiliated Hospital of Nantong University, Nantong 226001, Jiangsu, PR China; 2 Department of Chemotherapy, Affiliated Hospital of Nantong University, Nantong 226001, Jiangsu, PR China

**Keywords:** miR-95-3p, EMP1, gastric cancer, DDP resistance, PI3K/AKT

## Abstract

MicroRNAs (miRNAs) are thought to be involved in the development of cisplatin (DDP) resistance in gastric cancer (GC). Using RNA sequencing analysis (RNA-seq), we found that miR-95-3p is associated with DDP resistance in GC. We discovered that miR-95-3p is highly expressed in DDP-resistant GC tissues and cell lines (SGC7901/DDP and AGS/DDP). Furthermore, results from the BrdU and MTT assays indicated that miR-95-3p promotes GC cell proliferation. Additionally, data from transwell chamber assay, wound healing test and *in vivo* experiments illustrated that miR-95-3p can effectively promote invasion, migration and tumorigenic capacity, respectively, of DDP-resistant GC cells. Subsequently, results from dual luciferase assay and qRT-PCR collectively indicated that EMP1 is a target of miR-95-3p with inhibitory function through suppression of the EMT process and drug-resistance proteins. Furthermore, PI3K/AKT was identified as a downstream pathway of miR-95-3p, which promotes DDP resistance in GC. In summary, miR-95-3p helped develop DDP-resistance through down-regulation of EMP1 and increasing phosphorylation of the PI3K/Akt pathway in GC.

## INTRODUCTION

Gastric cancer (GC) is one of the most common malignant tumors, with the second highest incidence and the third highest mortality rate around the world [1, 2]. As GC patients are usually diagnosed at an advanced stage, chemotherapy is necessary for treatment of the disease [3]. Over the past decade, there has been great progress made in the diagnosis and treatment of GC; however, most patients remain incurable and have poor prognosis [4]. Among chemotherapeutic drugs, cisplatin (DDP) is the most commonly used and effective chemotherapeutic for treatment of GC [3]. DDP interferes with DNA replication, leading to death of rapidly proliferating cells. Initially, patients are highly responsive to cisplatin, but most patients eventually relapse and develop a strong resistance to DDP, which is a key obstacle when it comes to the effectiveness of GC treatment [5]. Studies have discovered that the mechanisms associated with DDP resistance include reduced drug uptake or enhanced drug efflux, altered molecular targets of anticancer drugs, enhanced DNA damage repair, and decreased expression of pro-apoptotic factors or increased expression of anti-apoptotic genes [6]. At the same time, genetic changes, including genetic mutations, translocations, deletions, and amplifications, and epigenetic changes, such as abnormal expression of microRNAs (miRNAs), are involved in the development of drug resistance [7]. Therefore, an in-depth understanding of the mechanism of DDP resistance and finding novel targets are very important for improving the prognosis of GC patients.

miRNAs are 20-22-nucleotide-long non-coding RNAs that have multiple regulatory functions and are associated with various physiological and pathological processes, including cancer development and progression. In general, miRNAs specifically target the 3’ untranslated regions (3’ UTRs) of specific messenger RNAs (mRNAs), thereby inhibiting expression of certain genes to regulate cellular processes [8, 9]. Recently, studies have revealed that miRNAs are involved in the development of drug-resistance. For instance, one study showed that miR-221 mediates Sorafenib resistance in hepatocellular carcinoma via modulation of caspase-3 [10]. In cisplatin-resistant ovarian cancer, levels of miR-137 are extremely low, which is related to high expression of c-Myc and EZH2. Interestingly, inhibition of the c-Myc-miR-137-EZH2 pathway was shown to make drug-resistant cells sensitive to cisplatin [11]. Furthermore, the miR-186-3p/EREG axis orchestrates tamoxifen resistance and aerobic glycolysis in ER-positive breast cancer [12]. Current research shows that miR-95-3p is dysregulated across numerous malignant tumors, including gastric cancer, osteosarcoma, prostate cancer, colon cancer, and liver cancer. In fact, miR-95-3p was found to be an indispensable molecular hub with regards to the processes of tumor proliferation, invasion and metastasis [13–17]. In addition, miR-95-3p has been shown to be closely related to radiotherapy sensitivity in non-small cell lung cancer, prostate cancer, and breast cancer [18, 19]. Using RNA sequencing analysis (RNA-seq), we found, for the first time, that miR-95-3p levels are significantly higher in cisplatin-resistant gastric cancer cells compared to cisplatin-sensitive cells. However, the specific role and underlying mechanism of miR-95-3p in the DDP-resistance of GC remain largely unknown.

In this study, we aimed to investigate the role and molecular mechanism of miRNA-95-3p in DDP resistance of gastric cancer. First, we found that miR-95-3p is highly expressed in the cisplatin-resistant gastric cancer cell line SGC7901/DDP. We also determined how low expression of miR-95-3p affects proliferation, migration, invasion, apoptosis and drug resistance in the cisplatin-resistant cells, and conducted relevant *in vivo* tumor-bearing studies. In addition, we studied the effect of miR-95-3p on the target gene EMP1 and related resistance pathways. These data are helpful in elucidating the function of miR-95-3p in DPP resistance of GC, and provide new ideas for drug resistance in treatment of GC.

## RESULTS

### Enhanced proliferative, invasive and migrative ability occurred in DDP-resistant GC cell line

**Figure 1:**
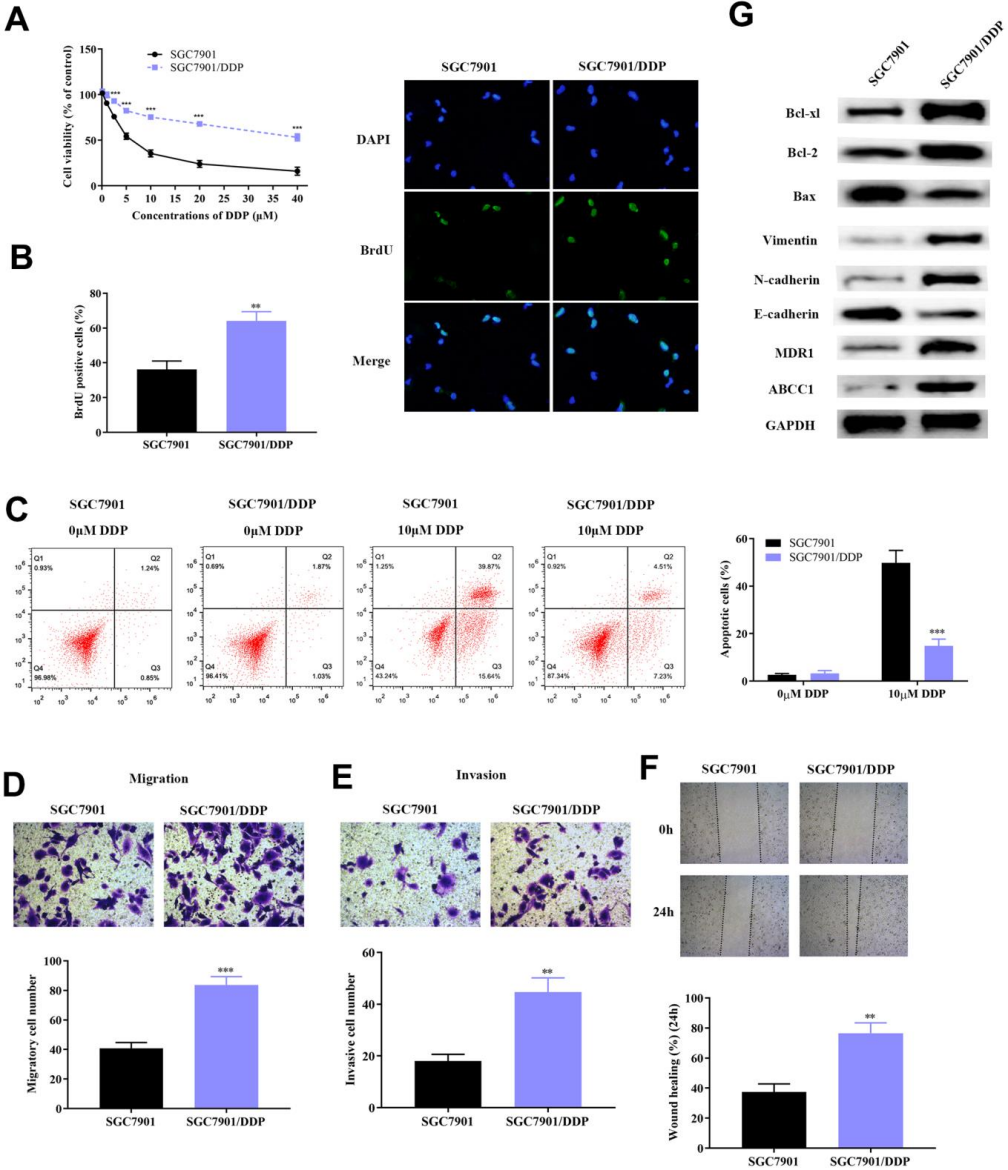
**Construction of DDP-resistant GC cell line (SGC7901/DDP).** (**A**) Cell viability assay demonstrated higher survival rate of SGC7901/DDP compared to normal SGC7901 cell line upon treatment with DDP. (**B**) BrdU assay showed that the DDP-resistant SGC7901 cell line had significantly higher survival rate compared to parental SGC7901. (**C**) Results from flow cytometry showed that the apoptotic rate of SGC7901/DDP cells was significantly lower compared to parental SGC7901. (**D**–**E**) Result of the transwell chamber assay indicated increased migration and invasion in SGC7901/DDP. (**F**) Result from the wound healing assay further verified the stronger invasive ability of SGC7901/DDP. (**G**) Result from western blot demonstrated lower apoptotic rate, stronger invasion and migration, increased drug resistance induced by higher expression of Bcl-2 family proteins, activation of EMT and overexpression of MDR1 and ABCC1 proteins, respectively. ***p<0.001, **p<0.01 compared to parental SGC7901.

The specific molecular mechanism that regulates DDP-resistance in GC still remains to be explored. In this study, the DDP-resistant GC cell lines (SGC7901/DDP and AGS/DDP) was constructed by continual treating cells with high concentrations of DDP. SGC7901 and AGS cells with DDP-resistance had stronger cell viability compared to the parental cell lines (Figure 1A and Supplementary Figure 1A). Further investigation of the proliferative capacity of SGC7901, AGS and SGC7901/DDP, AGS/DDP was determined using the BrdU assay. All GC cells were treated with DAPI, while BrdU represented the new synthetic GC cells (Figure 1B and Supplementary Figure 1B). After a 48-hour incubation, SGC7901/DDP and AGS/DDP cells demonstrated significantly high cell growth compared to the DDP-sensitive GC cells (SGC7901 and AGS), which indicated that resistance of DDP in GC cells improves cancer cell survival. We further studied GC cells progression, including proliferation, migration and invasion through flow cytometric assay, transwell assay and wound healing test, respectively. Firstly, SGC7901 and SGC7901/DDP were each divided into two groups. One group of SGC7901 and SGC7901/DDP was given 10 μM of DDP, while the other group of SGC7901 and SGC7901/DDP was given a same volume of PBS, which acted as the control group. Cellular apoptosis of SGC7901 and SGC7901/DDP were evaluated using flow cytometry (Figure 1C). At a concentration of 10 μM DDP, SGC7901/DDP cells demonstrated significantly lower apoptosis compared to SGC7901, which remained sensitive to DDP. A similar phenomenon was observed in the AGS/DDP cell line compared to the parental AGS line (Supplementary Figure 1C). Results of transwell chamber assay showed significantly higher metastatic and invasive cell quantities in SGC7901/DDP and AGS/DDP cell line (Figure 1D, 1E and Supplementary Figure 1D, 1E). Results from the wound healing test further validated these findings (Figure 1F and Supplementary Figure 1F). Furthermore, western blot analyses were conducted to identify the protein changes in DDP-resistant GC cells. The DDP-resistant SGC7901 had higher expression of anti-apoptosis proteins (Bcl-xl and Bcl-2), as well as up-regulation of EMT related proteins (Vimentin and N-cadherin) (Figure 1G).

MDR1 and ABCC1 were considered to be the two most important drug-resistance-related proteins in DDP-resistance across diverse cancers, including lung cancer, colorectal cancer and gastric cancer [20–22]. As a result, MDR1 and ABCC1 expression were detected in SGC7901/DDP cells. Our results show that MDR1 and ABCC1 are significantly overexpressed in DDP-resistant GC cells, compared to SGC-7901 cells. Taken together, these results indicated that DDP-resistant GC cells have higher cell proliferation, invasion and metastasis by activating apoptosis, EMT process and drug-resistance related proteins.

### miR-95-3p is up-regulated and associated with poor survival rate in GC patients

**Figure 2:**
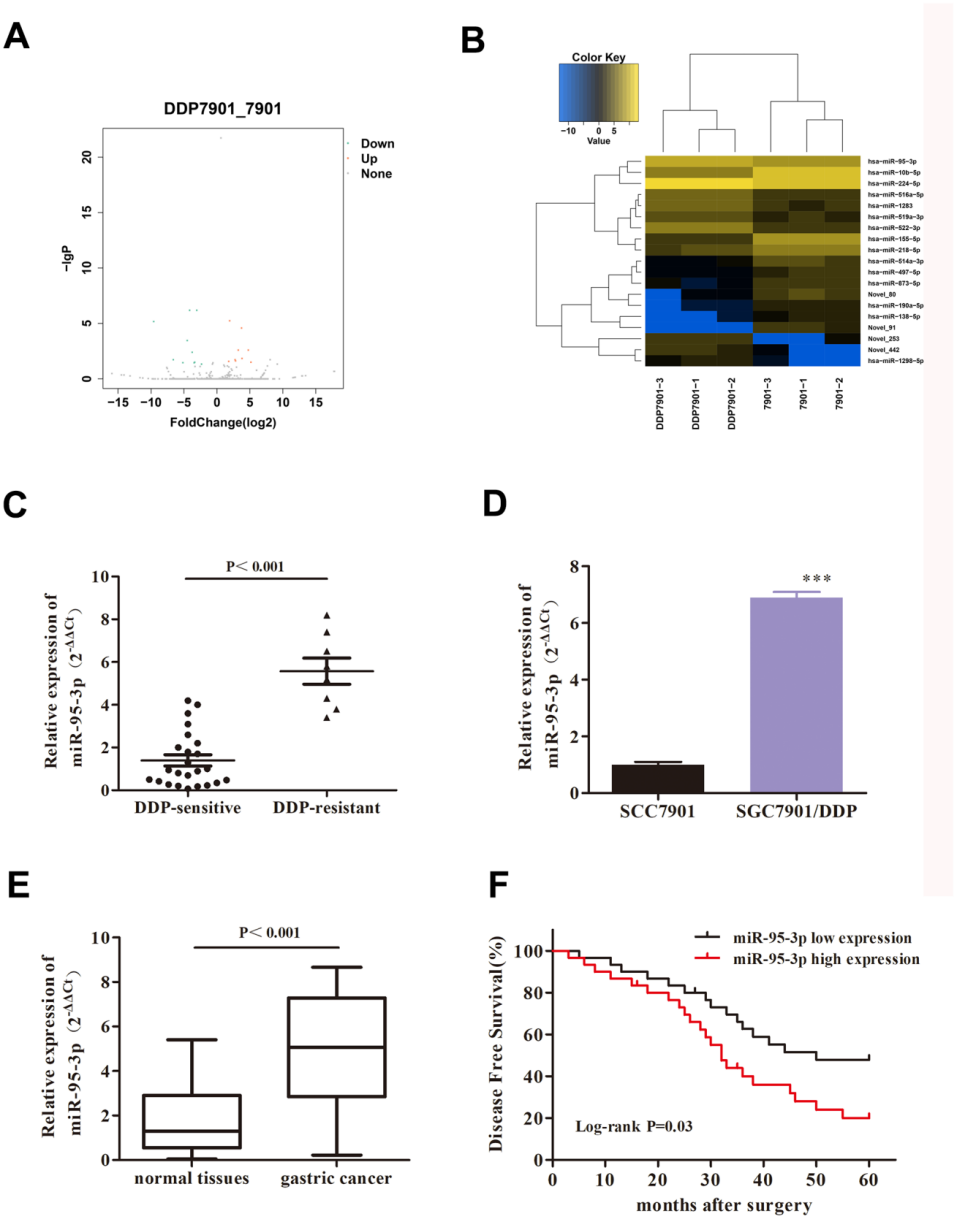
**MiR-95-3p is a pathologic gene in DDP-resistant GC.** (**A**) Volcano plot of the correlated gene of DDP-resistant SGC7901. (**B**) Heat map of DDP-resistant GC target gene. (**C**) Results from qRT-PCR indicated that miR-95-3p is overexpressed in DDP-resistant GC tissues compared to DDP-sensitive GC tissues. (**D**) qRT-PCR results indicated that miR-95-3p is highly expressed in DDP-resistant GC cell line. (**E**) qRT-PCR results indicated that expression of miR-95-3p is higher in gastric cancer tissues compared to paracancer tissues. (**F**) Low expression of miR-95-3p is associated with higher survival rate in gastric cancer patients. ***p<0.001, **p<0.01 compared to parental SGC7901.

According to the preset criteria of |log_2_(FC)|>1.0 and P-value <0.05, a total of 19 DE-miRNAs were identified, including 9 up-regulated miRNAs and 10 down-regulated miRNAs (Figure 2A, 2B) in the SGC7901/DDP cell line compared to normal SGC7901. Among the DE-miRNAs, hsa-miR-95-3p was found to be the highest DE-miRNA, and was subsequently selected as the focus of this study. As miR-95-3p was overexpressed in DDP-resistant GC tissues compared to DDP-sensitive tissues (Figure 2C), miR-95-3p might function as a contributor tumorigenesis and progression in GC. QRT-PCR results also confirmed that miR-95-3p is overexpressed in DDP-resistant cell lines and gastric cancer tissues (Figure 2D, 2E). Furthermore, gastric cancer patients with high miR-95-3p expression levels (top 50%) had shorter survival rate compared to GC patients with low miR-95-3p expression (bottom 50%) (Figure 2F). Consequently, miR-95-3p was chosen as the miRNA-of-interest for the subsequent experiments.

### Knockdown of miR-95-3p led to reduced cell growth, metastasis and invasion in gastric cancer

**Figure 3:**
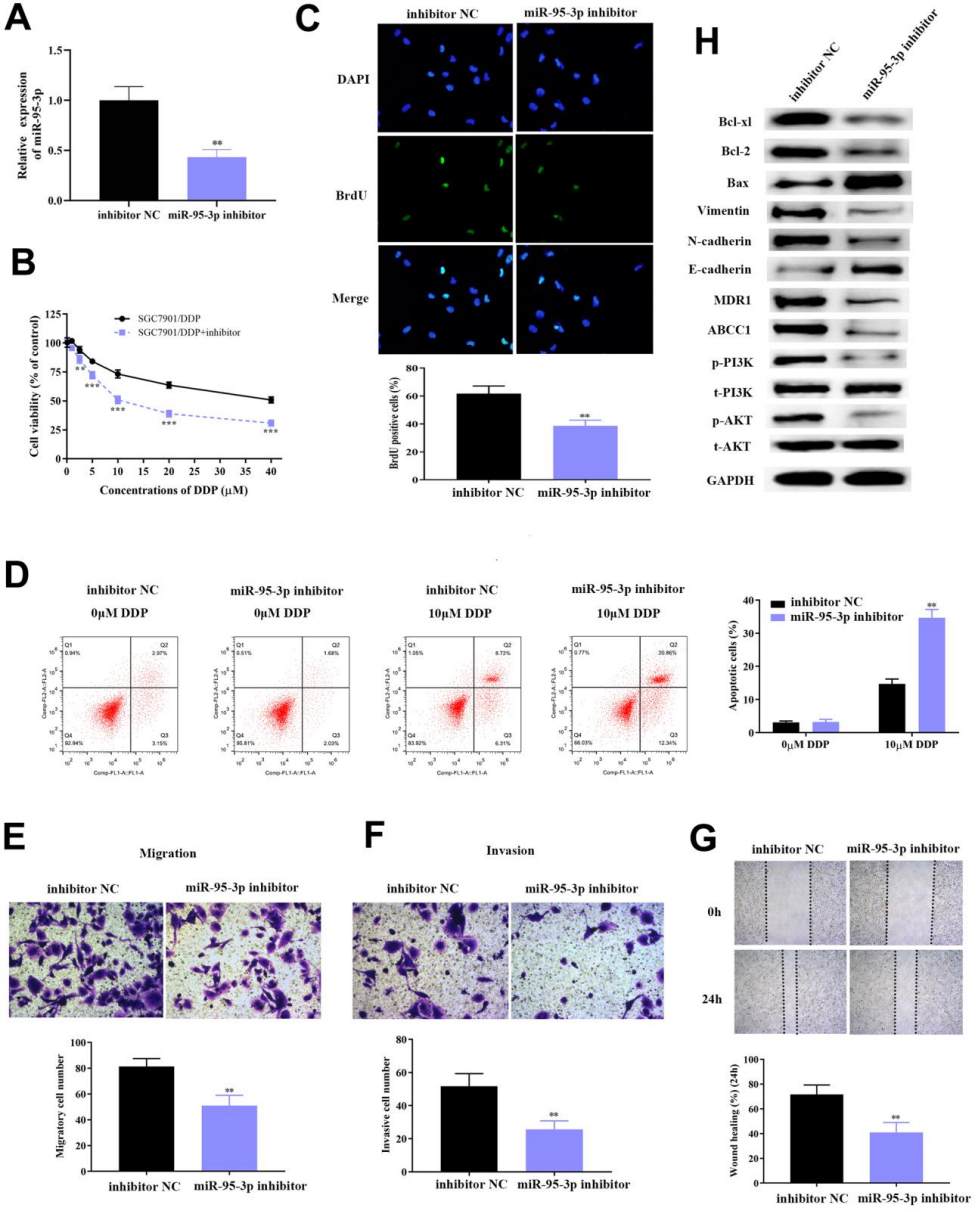
**Down-regulation of miR-95-3p leads to decreased cell viability, weakened invasion and migration, and enhanced cell apoptosis.** (**A**) Treatment with miR-95-3p inhibitor led to a decrease in expression of miR-95-3p. (**B**) MTT assay demonstrated that lower miR-95-3p levels led to weakened cell survival rate of DDP-resistant SGC7901. (**C**) BrdU assay results indicated that down-regulation of miR-95-3p reduced cell viability of DDP-resistant SGC7901. (**D**) Cellular apoptosis rate of DDP-resistant SGC7901. (**E**, **F**) Transwell assay results demonstrated lower cell invasion and metastasis of DDP-resistant SGC7901 upon down-regulation of miR-95-3p. (**G**) Wound healing assay suggested that down-regulation of miR-95-3p led to decreased invasion in DDP-resistant SGC7901. (**H**) Western blot assay helped examine the down-stream regulatory proteins of miR-95-3p. ***p<0.001, **p<0.01 compared to inhibitor NC.

Prior studies have reported that miR-95-3p is a carcinoma-promoting factor, as well as diagnostic and prognostic biomarker, for many cancers including hepatocellular carcinoma [23] and osteosarcoma [24]. As our previous studies indicate that miR-95-3p may be a favorable factor for tumorigenesis of GC, and so we subsequently down-regulated miR-95-3p in DDP-resistant SGC7901 and AGS cell lines using miR-95-3p inhibitor, and examined the expression of miR-95-3p through qRT-PCR. Results showed significantly higher miR-95-3p expression in SGC7901/DDP and AGS/DDP cell line versus cells treated with miR-95-3p inhibitor (Figure 3A and Supplementary Figure 2A). To determine whether there is a pathological relationship between the expression of miR-95-3p and GC progression (i.e. cell growth, metastasis and invasion), miR-95-3p inhibitor was used to treat SGC7901/DDP and AGS/DDP cells in order to knockdown miR-95-3p. The negative control of SGC7901/DDP and AGS/DDP cell line was treated with inhibitor NC. After 48 h, cells reached their ideal transfection for the continual experiments. Next, MTT and BrdU assay were conducted to determine the differences in proliferative capacity in SGC7901/DDP and AGS/DDP cells, as well as miR-95-3p down-regulated SGC7901/DDP and AGS/DDP cells. The results demonstrated that down-regulation of miR-95-3p in SGC7901/DDP and AGS/DDP cells induced a reduction in cell viability (Figure 3B and Supplementary Figure 2B) and cell proliferation (Figure 3C and Supplementary Figure 2C) compared to SGC7901/DDP and AGS/DDP with high expression of miR-95-3p. Then, 10 μM of DDP was administered to each group, and the same volume of PBS was utilized as negative control. Flow cytometry was conducted to further investigate the mechanism of how miR-95-3p regulates GC cell proliferation. Compared to the SGC7901/DDP and AGS/DDP cell line with high expression of miR-95-3p, down-regulation of miR-95-3p was able to reverse DDP-resistance in SGC7901/DDP and AGS/DDP cells, since knockdown of miR-95-3p led to significantly higher apoptosis rates (Figure 3D and Supplementary Figure 2D).

Subsequently, the effect of miR-95-3p in metastasis and invasion of DDP-resistant GC cell line (SGC7901/DDP and AGS/DDP) was studied using the transwell assay. Results indicated that down-regulation of miR-95-3p decreased migration and invasion of SGC7901/DDP and AGS/DDP when compared to SGC7901/DDP and AGS/DDP cells with high expression of miR-95-3p (Figure 3E, 3F and Supplementary Figure 2E, 2F). The wound healing test also validated the increased invasive ability in the miR-95-3p knockdown DDP-resistant GC cells (Figure 3G and Supplementary Figure 2G). Further exploration of the pathways involved was conducted by estimating the expression of related proteins through western blot.

### MiR-95-3p promotes GC cell progression via activating PI3K/AKT pathway, regulating apoptosis related proteins and stimulating EMT

The in-depth mechanism by which miR-95-3p promotes cell migration, invasion and proliferation were further investigated. Initially, we hypothesized that miR-95-3p may enhance metastatic and invasive ability of cells through epithelial-mesenchymal transition (EMT). Western-blot was used to determine the expression of EMT related proteins, including E-cadherin, N-cadherin and Vimentin. Results indicated that low expression of miR-95-3p led to an increase in expression of E-cadherin and a reduction in the levels of N-cadherin and Vimentin (Figure 3H), which validated our hypothesis that miR-95-3p promotes migration and invasion through EMT. Additionally, we determined the expression of drug resistance-related proteins (MDR1, ABCC1) and found them to be decreased (Figure 3H).

Next, the quality of proteins within the PI3K/AKT pathway was also evaluated. Knockdown of miR-95-3p led to down-regulation of phosphorylated PI3K and AKT in comparison to SGC7901/DDP (Figure 3H). Additionally, apoptosis-related proteins (i.e. Bax, Bcl2 and Bcl-xl) were also determined. Bax expression was significantly higher, while Bcl2 and Bcl-xl were lower in SGC7901/DDP cells treated with miR-95-3p inhibitor (Figure 3H). Consequently, these results indicated that miR-95-3p promotes cell proliferation by stimulating the PI3K/AKT pathway and prevents cellular apoptosis through apoptosis-related proteins and stimulating EMT.

### Inhibition of miR-95-3p alleviates tumor growth *in vivo*

**Figure 4:**
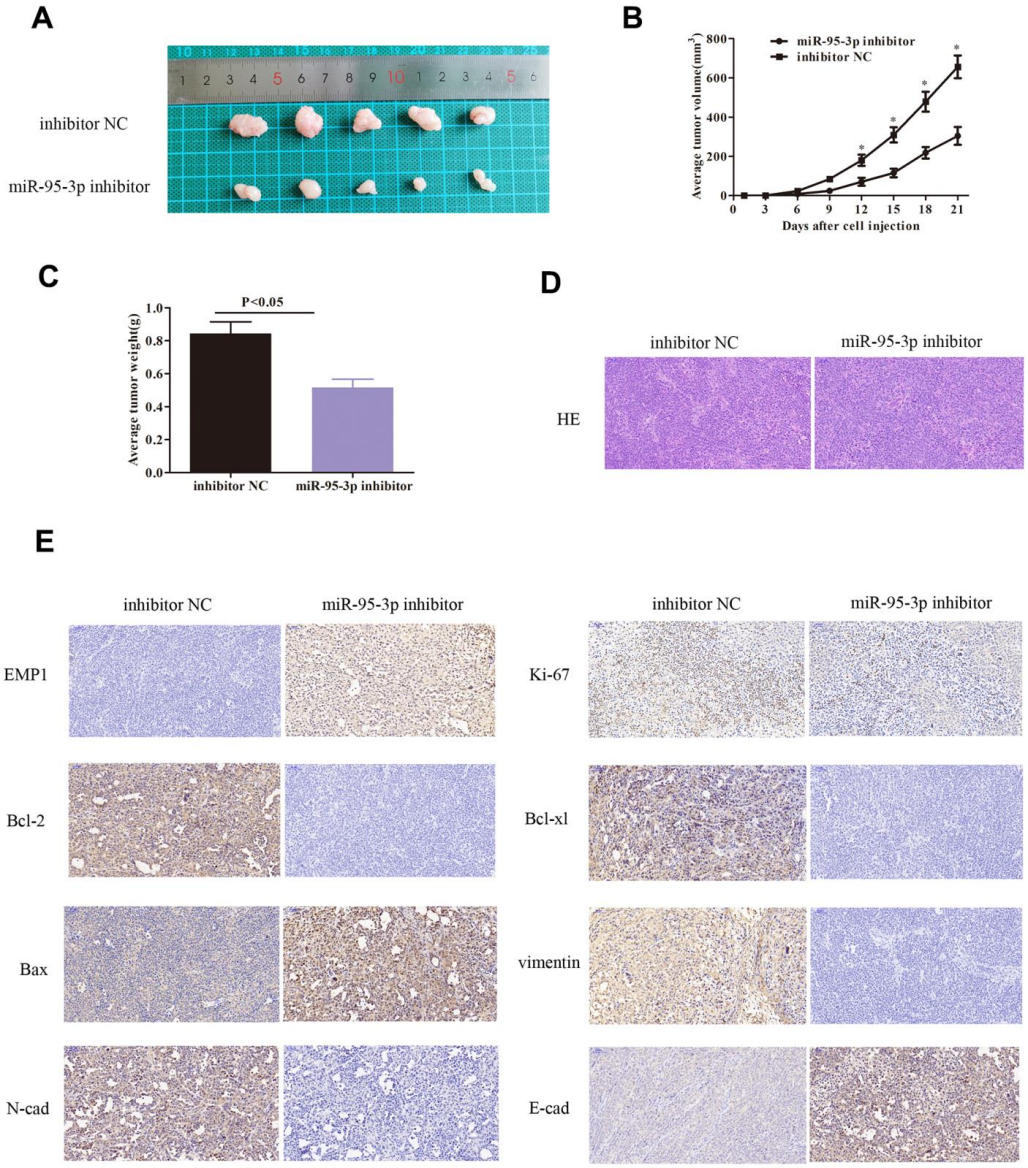
**Down-regulation of miR-95-3p induced slower tumor growth *in vivo* via decreased expression of oncogenes, inhibiting the EMT process, and down-regulation of drug-resistance proteins.** (**A**) Tumor volume was lower upon treatment with miR-95-3p inhibitor after 21 days of treatment. (**B**) Tumor volume escalated at a slower rate in the miR-95-3p inhibitor treated group. (**C**) Lower tumor weight occurred in the miR-95-3p inhibitor-treated group. (**D**) H&E staining of the tumor. (**E**) Immunohistochemical staining result of different proteins.

Further investigation of miR-95-3p *in vivo* was conducted using BALB/c nude mice inoculated with DDP-resistant gastric cancer cells (SGC7901/DDP) that were transfected with miR-95-3p inhibitor or inhibitor NC. After 21 days of observation, we recorded the weight of each mice and the volume of each tumor. Our results indicated that down-regulation of miR-95-3p in DDP-resistant gastric cancer curtailed tumor growth *in vivo* as tumor volume was lower small compared to control group (Figure 4A–4C). Next, gastric tumor tissues were resected and stained using hematoxylin and eosin (H&E) (Figure 4D). Furthermore, in order to investigate the expression of pathologically correlated proteins, we performed immunohistochemical staining. Cells treated with miR-95-3p inhibitor exhibited up-regulation of EMP1, Bax and E-cadherin, and downregulation of Bcl-2 and Bcl-xl, Vimentin, N-cadherin and Ki-67, suggesting that miR-95-3p inhibits DDP-resistance by preventing EMT process (Figure 4E).

### EMP1 was identified as a target gene of miR-95-3p

**Figure 5:**
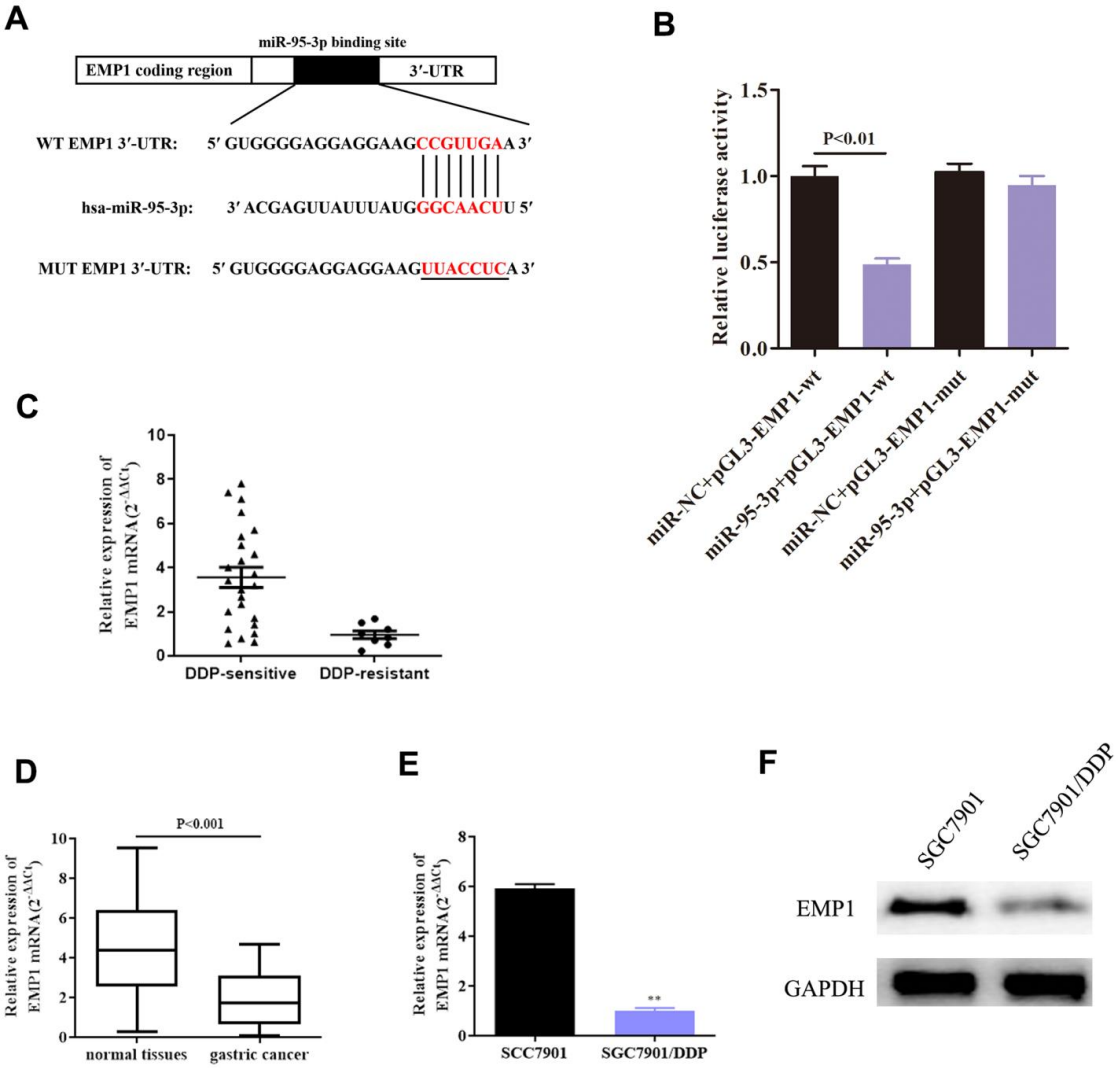
**EMP1 and miR-95-3p directly interact.** (**A**) *In silico* prediction through the miRanda database demonstrated the presence of a binding site of miR-95-3p on EMP1 sequence. (**B**) Results from dual luciferase activity validate direct interaction between miR-95-3p and EMP1. (**C**) QRT-PCR results indicate EMP1 is lowly expressed in DDP-resistant GC tissues. (**D**) QRT-PCR suggests that EMP1 is lowly expressed in GC tissues compared to normal adjacent tissues. (**E**, **F**) QRT-PCR and western blot assay suggest that EMP1 is down-regulated in DDP-resistant SGC7901 cell line. **p<0.01 compared to parental SGC7901.

Further exploration of the downstream target genes of miR-95-3p was conducted using bioinformatic analyses. Starbase, DIANA and TargetScan were used to predict biological binding information. Results indicated that there was a binding site to EMP1 on the miR-95-3p sequence (Figure 5A). In order to validate this prediction, we conducted a dual-luciferase assay to verify direct binding between EMP1 and miR-95-3, results of which indicated that EMP1 was indeed a target gene of miR-95-3p (Figure 5B). Next, we studied the relationship between EMP1 and miR-95-3p using qRT-PCR. The results showed that EMP1 is expressed at low levels in DDP-resistant GC tissues, while DDP-sensitive GC tissues have high expression of EMP1 (Figure 5C). Additionally, EMP1 is downregulated in gastric cancer tissues compared to normal tissues (Figure 5D). Furthermore, EMP1 was found to be lowly expressed in DDP-resistant GC cell line (Figure 5E). Western blot findings further confirmed this finding (Figure 5F).

### EMP1 suppresses DDP-resistant GC cell progression via PI3K/Akt signaling

**Figure 6:**
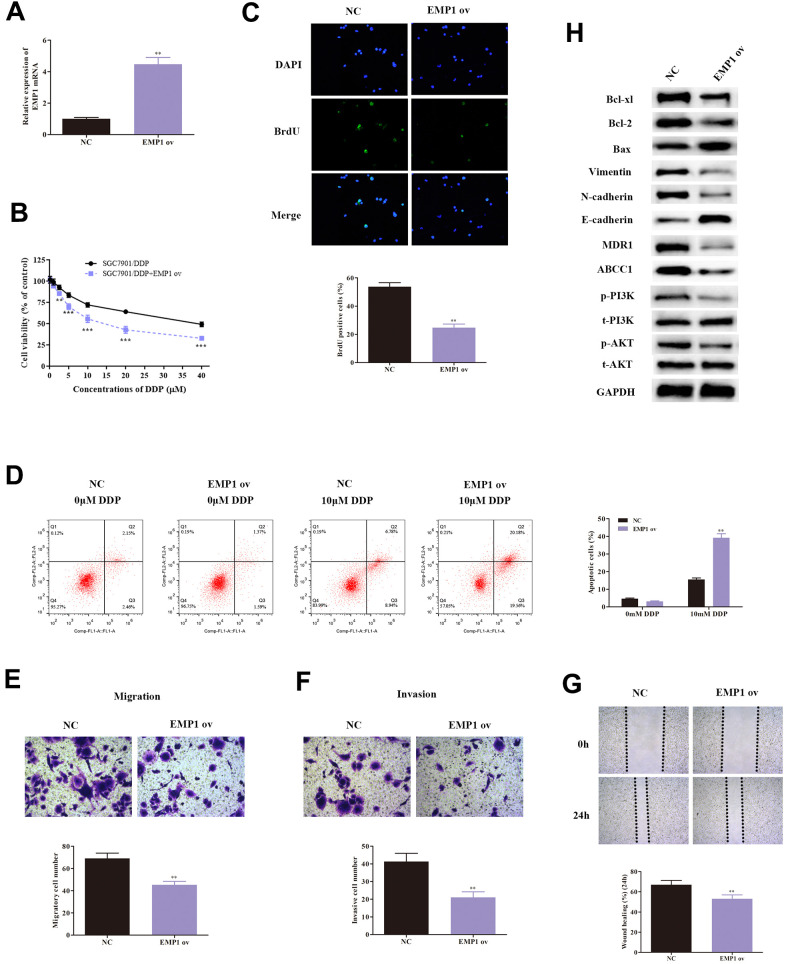
**Over-expression of EMP1 leads to decreased cell viability, weakened invasion and migration ability, and enhanced cellular apoptosis.** (**A**) Transfection of EMP1 ov vectors increased expression of EMP1. (**B**) MTT assay demonstrated that overexpressed EMP1 induced weakened cell survival rate of DDP-resistant SGC7901 cells. (**C**) Result of the BrdU assay indicated that overexpressed EMP1 lowered cellular viability of DDP-resistant SGC7901. (**D**) Cellular apoptosis rate of DDP-resistant SGC7901 cells with different treatments. (**E**, **F**) Results from transwell assay demonstrated lower cell invasion and metastasis of DDP-resistant SGC7901 upon up-regulation of EMP1. (**G**) Wound healing assay indicated up-regulation of EMP1 leads to decreased invasive ability in DDP-resistant SGC7901. (**H**) Western blot assay helped examine the down-stream regulatory proteins of EMP1. ***p<0.001, **p<0.01 compared to NC group.

As EMP1 was proven to be a target of miR-95-3p, we investigated the effect of EMP1 in SGC-7901/DDP cells. We validated the transfection efficiency of EMP1 overexpression using qRT-PCR, which showed significantly higher EMP1 expression in SGC7901/DDP cells treated with EMP1 ov (Figure 6A). Next, MTT assay and BrdU assay were conducted to determine the differences in proliferative capacity in SGC7901/DDP cells. The results demonstrated that up-regulation of EMP1 in SGC7901/DDP cells led to a reduction in cell viability (Figure 6B) and less cell proliferation (Figure 6C). High expression of EMP1 also significantly increased apoptosis, induced by DDP (Figure 6D).

Subsequently, transwell assay showed that up-regulation of EMP1 decreased migratory and invasive capability of SGC7901/DDP cells (Figure 6E, 6F). The wound healing test also validated high level of EMP1 lead to increased invasive ability in DDP-resistant GC cells (Figure 6G).

Western-blot was conducted to examine the expression of EMT-related proteins, including E-cadherin, N-cadherin and Vimentin. Results indicated that high expression of EMP1 led to an increase in expression of E-cadherin, and a decrease in levels of N-cadherin and Vimentin (Figure 6H). These findings validated our hypothesis that EMP1 suppressed migration and invasion through EMT. Additionally, the expression of drug-resistance related proteins (MDR1, ABCC1) were determined and found to be reduced by up-regulation of EMP1 (Figure 6H).

Next, we also assessed the expression of related proteins in the PI3K/AKT pathway. Overexpression of EMP1 led to a down-regulation of phosphorylation of PI3K and AKT compared to SGC7901/DDP (Figure 6H). Additionally, apoptosis-related proteins, including Bax, Bcl2 and Bcl-xl, were also tested. Levels of Bax were significantly higher, while Bcl2 and Bcl-xl were significantly lower in SGC7901/DDP with EMP1 ov (Figure 6H). Consequently, the results suggest that EMP1 can suppress DDP-resistant GC cells progression through inactivation of the PI3K/AKT pathway.

### Up-regulation of EMP1 reversed DDP-resistance and curtailed tumor progression of gastric cancer

**Figure 7:**
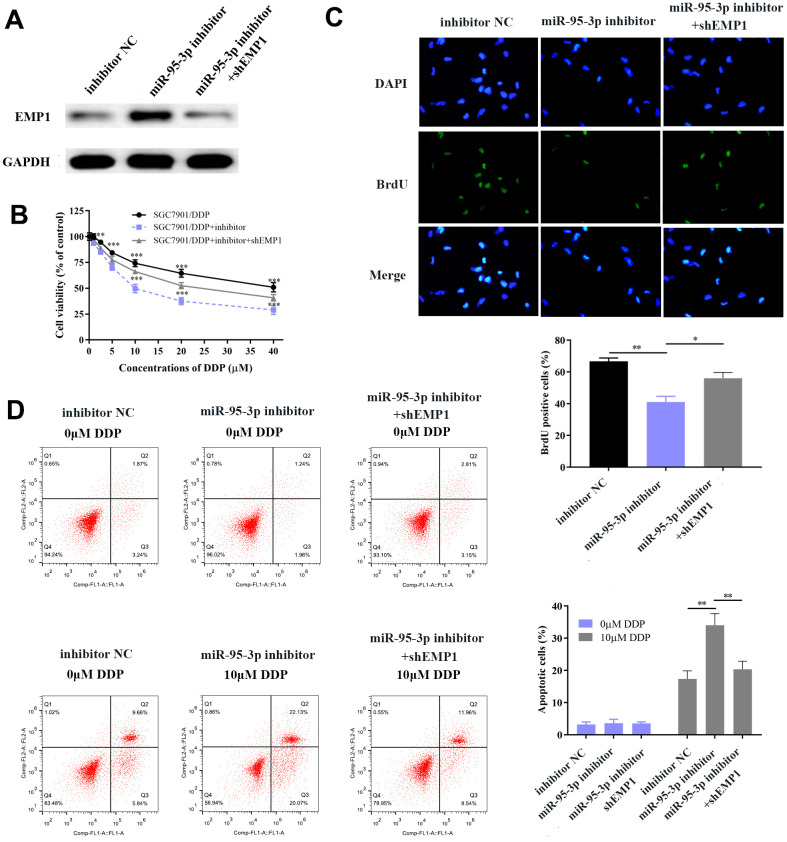
**EMP1 promotes GC cellular apoptosis and prevents cell proliferation.** (**A**) Western blot showed that down-regulation of miR-95-3p led to up-regulation of EMP1. (**B**) Down-regulation of EMP1 led to higher cell viability when compared to the miR-95-3p inhibitor group. (**C**) BrdU assay indicated that knockdown of EMP1 led to higher cell survival rate compared to miR-95-3p inhibitor treated group. (**D**) Flow cytometry demonstrated that up-regulation of EMP1 (miR-95-3p inhibitor) led to higher apoptosis when compared to the EMP1 knockdown group. ***p<0.001, **p<0.01.

**Figure 8:**
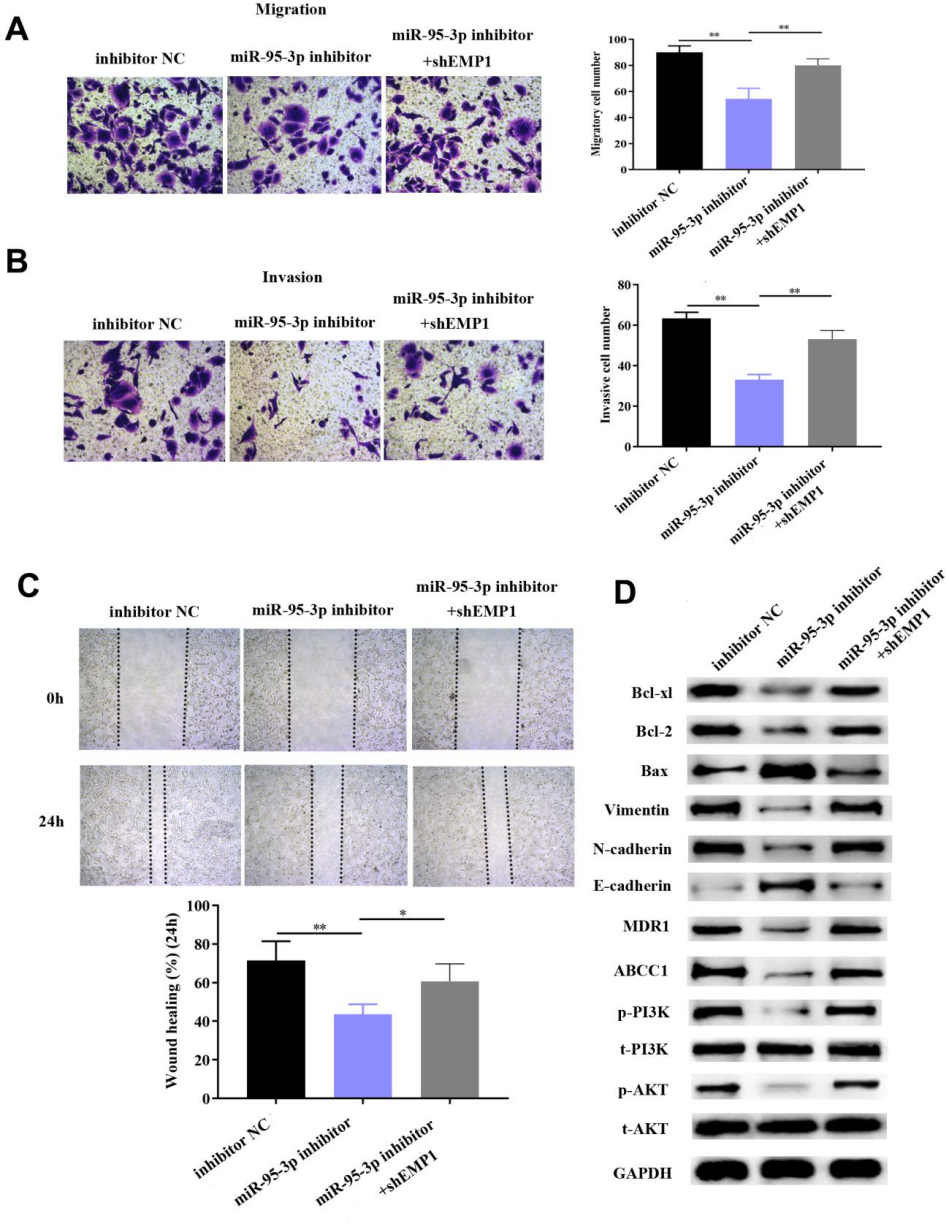
**Down-regulation of EMP1 led to improved cell migration and invasion via activation of the EMT process and PI3K/AKT pathway.** (**A**, **B**) Transwell chamber assay demonstrated that knockdown of EMP1 led to increased invasion and migration compared to the miR-95-3p inhibitor group. (**C**) Wound healing assay further verified that knockdown of EMP1 led to increased cell migration compared to the miR-95-3p inhibitor group. (**D**) Western blot assay indicated that knockdown of EMP1 induced up-regulation of neoplastic proteins (Bcl-xl and Bcl-2), activation of EMT and PI3K/AKT pathway compared to the miR-95-3p inhibitor group. ***p<0.001, **p<0.01.

Next, we explored the specific function by which EMP1 restores DDP-resistance of GC cells. Small interference RNA-EMP1 (sh-EMP1) and miR-95-3p inhibitor were co-transfected into SGC7901/DDP in order to down-regulate EMP1 expression. MiR-95-3p inhibitor and negative control were used to construct an EMP1-upregulated and control cell line, respectively. Western blotting was used to verify the transfection efficiency of sh-EMP1 (Figure 7A). MTT assay and BrdU assay were utilized to examine cell viability and proliferative capacity, the results of which showed that down-regulation of EMP1 in the SGC7901/DDP group had significantly higher cell viability compared to miR-95-3p inhibitor treated group (Figure 7B, 7C). Additionally, cellular apoptosis rate was decreased when 10μM of DDP was administered to the EMP1 silenced group (Figure 7D). The sensitivity toward DDP was estimated through testing the rate of apoptosis using flow cytometry, results of which suggested that lower apoptosis in sh-EMP1-transfected SGC7901/DDP cells treated with DDP. Overall, this suggests that EMP1 can reverse DDP-resistance in gastric cancer. Additionally, transwell assay demonstrated that silencing of EMP1 resulted in stronger migration and invasion in DDP-resistant GC cells (Figure 8A, 8B), and findings from the wound healing test further verified the migration preventing function of EMP1 (Figure 8C).

We hypothesized that EMP1 is a mediating factor of downstream pathways, including PI3K/AKT and EMT. Thus, we used western blot to determine the expression of related proteins. Results showed silencing of EMP1 led to a down-regulation of E-cadherin and up-regulation of N-cadherin and Vimentin, which indicates that EMT becomes stimulated upon knockdown of EMP1. Subsequently, apoptosis-related proteins (i.e. Bcl2 and Bcl-xl) were found up-regulated in SGC7901/DDP cells with knockdown of EMP1, which illustrated that silencing of EMP1 can preclude apoptosis by enhancing the expression of apoptosis-related proteins (Figure 8D). Finally, proteins involved in the PI3K/AKT pathway (i.e. p-PI3K and p-AKT) were found to be overexpressed in the EMP1 knockdown group (Figure 8D), which validated that EMP1 is a crucial factor in curtailing activation of the PI3K/AKT pathway. Also, expression of drug resistance related proteins (MDR1, ABCC1) were determined and found to be increased after knockdown EMP1 (Figure 8D).

### PI3K/AKT is a positive regulatory downstream pathway mediated by miR-95-3p

**Figure 9:**
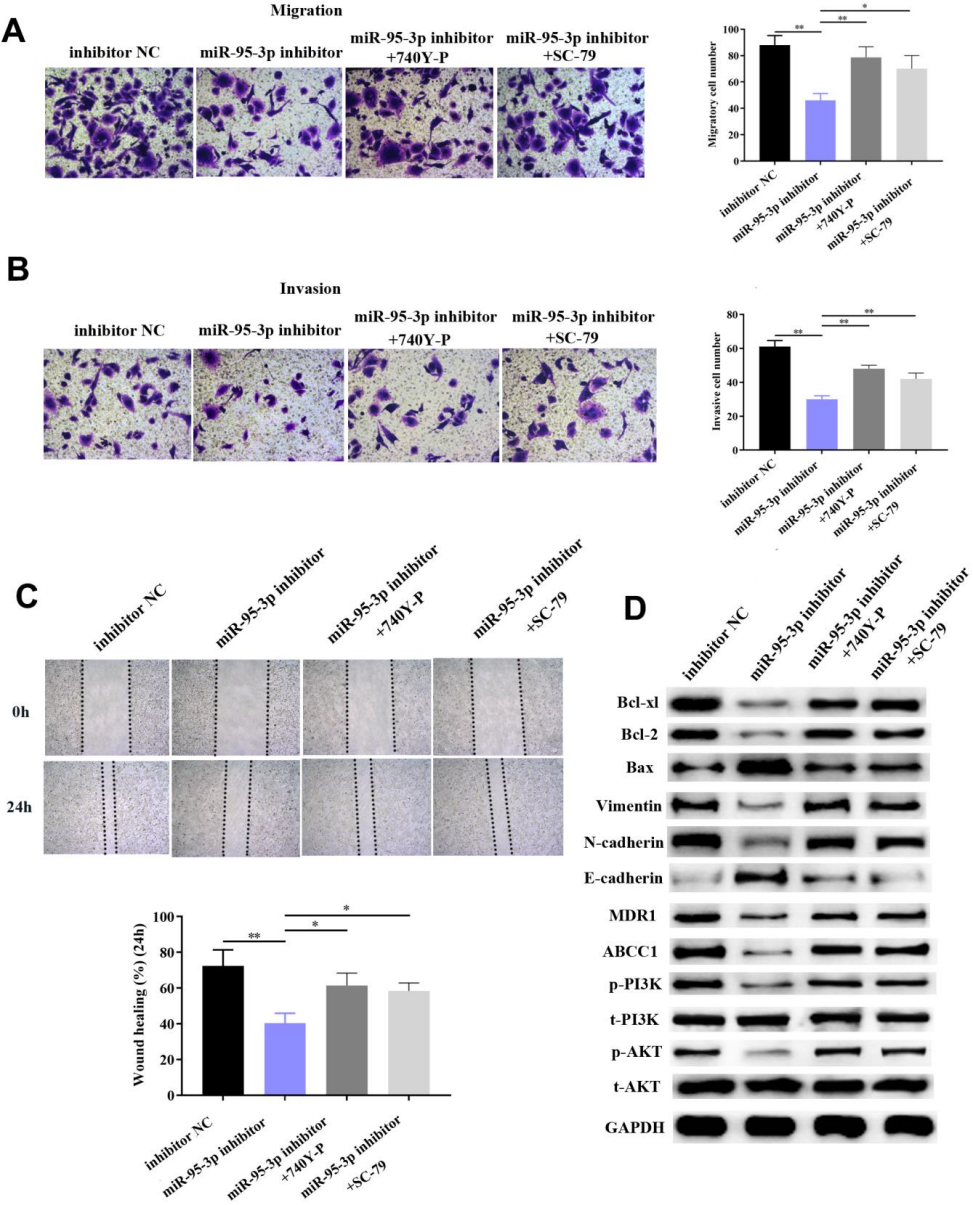
**Activation of the PI3K/AKT pathway impaired DDP sensitivity among GC cells.** (**A**) Activation of the PI3K/AKT pathway does not affect expression of EMP1. (**B**) Use of PI3K/AKT activation impaired GC cells’ sensitivity toward DDP compared to the miR-95-3p inhibitor treated group. (**C**) Result from the BrdU assay further indicated that PI3K/AKT activator significantly enhanced DDP-resistant cell survival rate compared to the miR-95-3p inhibitor-treated group. (**D**) Results from flow cytometry demonstrated that PI3K/AKT activator could conspicuously decrease cellular apoptosis rate compared to the miR-95-3p inhibitor treated group. ***p<0.001, **p<0.01.

**Figure 10:**
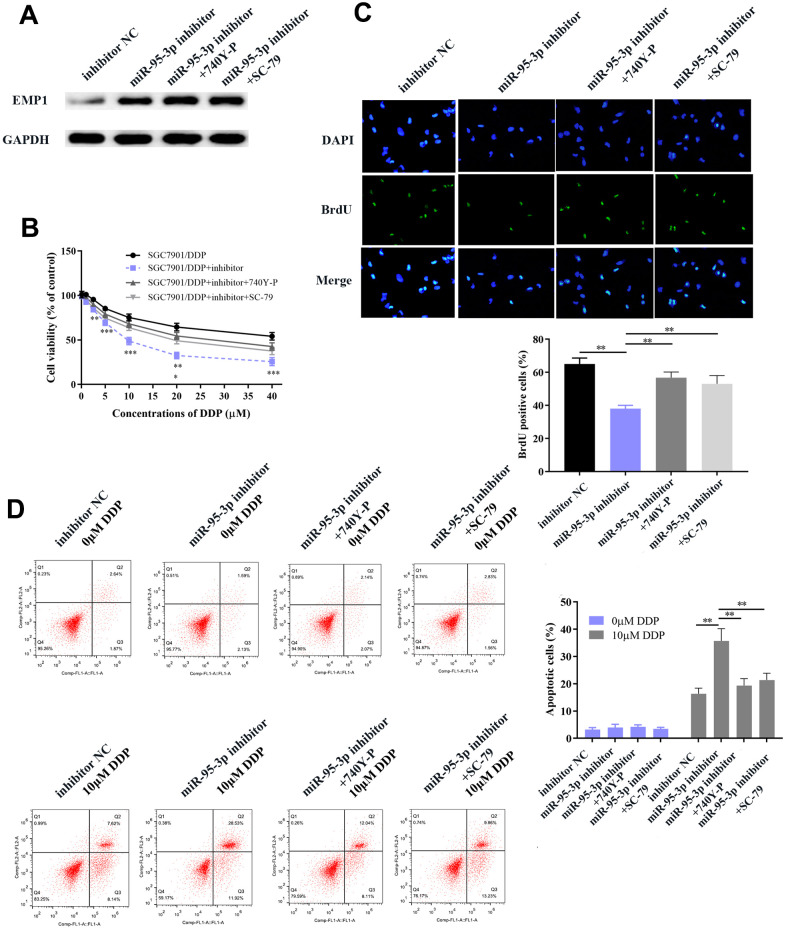
**Activation of the PI3K/AKT pathway promoted DDP-resistant SGC7901 cell proliferation, invasion and migration through up-regulation of related neoplastic proteins (Bcl-xl and Bcl-2), EMT-related proteins and drug resistance-related proteins.** (**A**, **B**) Transwell chamber assay indicated increased invasion and migration in the PI3K/AKT activator-treated group compared to the miR-95-3p inhibitor treated group. (**C**) Wound healing assay indicated that the PI3K/AKT activator significantly improved DDP-resistant SGC7901 cellular invasion. (**D**) Western blot results demonstrated that the use of a PI3K/AKT activator promoted oncogenic expression, and increase of EMT process-related proteins and drug resistance-related proteins. ***p<0.001, **p<0.01.

In order to further verify that the PI3K/AKT downstream pathway was positively regulated by miR-95-3p, cells were co-transfected with a PI3K agonist (740Y-P) or AKT agonist (SC79) with miR-95-3p inhibitor. After co-transfection with 740YP or SC79, EMP1 protein expression was not significantly changed compared to miR-95-3p inhibitor group (Figure 9A). Results from the MTT and BrdU assay demonstrated that down-regulation of miR-95-3p can alleviate cell viability and proliferation, while treatment of 740Y-P or SC79 induced the opposite effect, which promoted cell growth of DDP-resistant gastric cancer (Figure 9B, 9C). Subsequently, flow cytometry further confirmed the function of 740Y-P or SC79 in restoring DDP resistance in GC cells. (Figure 9D). Previous experiments confirmed that down-regulation of miR-95-3p weakens the metastatic and invasive capacity of DDP-resistant GC cells. However, administration of PI3K or AKT agonist (740Y-P, SC79) led to a surge in the number of migrated and invasive cells (Figure 10A, 10B). Simultaneously, results from the wound healing test further verified the enhanced migratory capacity (Figure 10C). The expression of drug resistance related proteins (i.e. MDR1 and ABCC1) of miR-95-3p was determined through western blot. Results indicated that activation of the PI3K/AKT pathway by 740Y-P or SC79 in SGC7901/DDP cells with miR-95-3p knockdown induced an increase in MDR1 and ABCC1 expression (Figure 10D), which further validated that miR-95-3p contributes to the development of DDP resistance in gastric cancer. Furthermore, the expression of PI3K/AKT pathway related proteins (p-PI3K and p-AKT) were examined. We found that expressions of these proteins were reduced when miR-95-3p was down-regulated, while overexpression of 740Y-P or SC79 alongside treatment with miR-95-3p inhibitor led to an increase in the expression quantities of p-PI3K and p-AKT. The expression of EMT related proteins (N-cadherin and vimentin) and apoptosis related proteins (Bcl2 and Bcl-xl) were also evaluated and were found to be down-regulated when treated with miR-95-3p inhibitor, while 740Y-P or SC79 served the opposite function. These results collectively suggest that miR-95-3p is a crucial regulatory factor in enhancing the drug resistance and tumorigenesis of gastric cancer with resistance to DDP.

## DISCUSSION

Gastric cancer is a devastatingly lethal disease that has a low 5-year survival rate of 20.8% -36.8%, and has been ranked as the third common cause for cancer-related mortality [25, 26]. The biomarkers that are currently used for a clinical diagnosis, including carcinoembryonic antigen (CEA) and carbohydrate antigen (CA), are deficient in sensitivity and specificity, leading to an early gastric cancer diagnosis rate of lower than 15% [27, 28]. Therefore, there is a need to identify more efficient tumor biomarkers for early diagnosis of gastric cancer. Nowadays, although cisplatin (DDP)-based chemotherapy has become the predominant approach for treatment of gastric cancer, the clinical applications are limited due to the development of drug resistance, which inevitably induces poor prognosis or even death [29]. Hence, understanding the in-depth mechanism of drug resistance and development of drug resistance-reversing approaches are significant problems that scientists need to work on.

The mechanisms of cisplatin resistance in gastric cancer are complicated and still need to be explored. MicroRNAs (miRNAs) are increasingly becoming the research focus of carcinoma diagnosis and therapies as miRNAs have been identified as playing pathological roles in the tumorigenesis of various cancers, including breast cancer, hepatocellular carcinoma, colon cancer and lung cancer [30–33]. Dysregulation of miRNAs is clinicopathologically correlated to growth signal disorder, immune escape, angiogenesis, tumor tissue invasion and metastasis [34]. Different miRNAs participate in various processes of tumorigenesis of gastric cancer through mediating specific target genes [35, 36]. MiR-95-3p has been reported to be an oncogene that promotes proliferation and invasion. For example, miR-95-3phelps glioma cells evade apoptosis by down-regulating its downstream target gene CUGBP-and-ETR-3-like family 2 (CELF) [17]. Furthermore, prior studies have revealed that miR-95-3p is a biomarker for diagnosis and prognosis in osteosarcoma [24]. Furthermore, up-regulation of miR-95-3p enhances tumor progression through down-regulation of p21 [23].

In this study, we identified miR-95-3p as a DE-miRNA with significantly differential expression between DDP-sensitive GC cell line (SGC7901) and DDP-resistance GC cell line (SGC7901/DDP), and was therefore chosen as our research subject for subsequent investigation. Next, we analyzed the expression of miR-95-3p in DDP-resistant GC tissues, DDP-sensitive GC tissues and normal tissue, and found that miR-95-3p was overexpressed in GC, which was validated by genetic sequencing. To further understand the specific functions of miR-95-3p in regulating GC progression, miR-95-3p inhibitor was transfected into SGC7901/DDP and AGS/DDP cell lines to down-regulate miR-95-3p expression. Downregulation of miR-95-3p led to a reduction in cell proliferation, migration and invasion, as observed through MTT assay, BrdU assay, transwell assay and wound healing test, respectively. These results indicated that knockdown of miR-95-3p in DDP-resistant GC cells inhibits tumor progression. Western-blot assay for evaluation of proteins involved in drug resistance, EMT, apoptosis and PI3K/AKT pathway revealed that miR-95-3p improved GC progression by up-regulating MDR1 and ABCC1, stimulating the EMT process, increasing expression of Bcl2 and Bcl-xl and activating the PI3K/AKT pathway. Further exploration of miR-95-3p was conducted *in vivo* using the nude mice inoculated with DDP-resistant GC. After 21 days of observation, nude mice treated with miR-95-3p inhibitor had the smallest tumor swelling with a stable tiny tumor volume and mouse weight. Immunohistochemistry was performed, which indicated that there was less tumor progression in the miR-95-3p treated group compared to the control group.

It has been suggested that epithelial membrane protein 1 (EMP1) is a potential downstream target gene of miR-95-3p. Studies have found that EMP1 serves as a tumor suppressor in multiple carcinomas. It has been reported that EMP1 expression is significantly lower in laryngeal carcinoma compared to normal adjacent tissues [37]. Furthermore, EMP1 was disclosed as having a function to prevent nasopharyngeal cancer proliferation and metastasis by enhancing apoptosis and reducing angiogenesis [38]. Additionally, EMP1 has been shown to be a biomarker in tumors resistant to gefitinib, which suggests that EMP1 may play a functional role in regulating the development of drug resistance [39]. Next, miRanda, PicTar and TargetScan were utilized to identify a continuous binding sequence of miR-95-3p on EMP1. In order to verify this prediction, a dual-luciferase assay was performed, and was later discovered that there was direct binding between miR-95-3p and EMP1. EMP1 has been reported to suppress tumor progression across various cancer types, including nasopharyngeal cancer, oral squamous cell carcinoma and prostate cancer [38, 40, 41]. On the contrary, EMP1 has been shown to be involved in cancer progression, including ovarian cancer and pediatric leukemia [42, 43], which suggests that the anti-tumor or tumor-promoting action of EMP is dependent on the type of cancer. Some studies have demonstrated that EMP1 can regulate the PI3K/AKT pathway [44, 45]. As a result, additional investigation of EMP1 on SGC-7901/DDP cells was conducted. Firstly, it was revealed that EMP1 is negatively regulated by miR-95-3p. Next, we discovered that transfection of si-EMP1 into the SGC7901/DDP cell line with miR-95-3p exerted a profound effect on the progression of gastric cancer. Results indicated that knockdown of EMP1 strengthened cell proliferation, metastasis and invasion of GC. Furthermore, findings from western-blot indicated that down-regulation of EMP1 led to higher expression of apoptosis related proteins (Bcl2 and Bcl-xl), EMT related proteins (N-cadherin, Vimentin), drug resistance proteins (MDR1, ABCC1) and PI3K/AKT related proteins (PI3K and p-PI3K). These results suggest that EMP1 can prevent gastric cancer progression by suppressing the EMT process, PI3K/AKT pathway, and promoting apoptosis. An in-depth exploration of the relationship between miR-95-3p and PI3K/AKT pathway was conducted by treating the SGC7901/DDP cell line with miR-95-3p inhibitor and a PI3K/AKT agonist (740Y-P, SC79). Results indicated that upon administration of the PI3K/AKT agonist, the PI3K/AKT pathway, which had been inhibited by miR-95-3p inhibitor, was reactivated. Furthermore, the DDP-sensitivity brought by miR-95-3p was weakened by use of the PI3K/AKT agonist.

## CONCLUSIONS

Overall, we conclude that miR-95-3p help develop resistance to DDP and promote cell proliferation, migration, and invasion by down-regulating EMP1 and increasing the phosphorylation of PI3K/AKT pathway in gastric cancer.

## MATERIALS AND METHODS

### Patients and samples

Tissue samples were collected, prior to initiating DDP-based therapy, from biopsies of advanced gastric cancer patients (n=32; cohort 1) and from R0 excision patients (n=60; cohort 2), between January 2013 and December 2014. Tumor response to chemotherapy was assessed using a 3-dimensional volume reduction rate or tumor response rate (radiologic assessment), and evaluated according to the Response Evaluation Criteria in Solid Tumors (RECIST) guidelines. Patients with deterioration of symptoms, appearance of new lesions, or radiologic assessment of ≥25% tumor regrowth in validation phase were assigned to the DDP-resistant group, while the remaining patients were assigned to the DDP-sensitive group. Disease-free survival (DFS) was defined as the time period between gastrectomy (R0 excision) and time to either disease recurrence or disease-associated death. This study was granted approval by the Ethics Committee of the Affiliated Hospital of Nantong University.

### Cell lines and incubational conditions

Human gastric cell lines (SGC7901, AGS) were purchased through the Type Culture Collection of the Chinese Academy of Sciences, located in Shanghai, China. The cells were grown in 1640 medium (Gibco, Gaithersburg, MD, USA) containing 10% fetal bovine serum (FBS, Gibco) and incubated at 37° C and 5% CO_2_. The cisplatin-resistant gastric cancer cell lines SGC-7901/DDP and AGS/DDP were produced from the parental SGC-7901 and AGS cells through persistence gradient exposure to cisplatin for approximately 12 months, and was subjected to increasing concentrations of cisplatin (Sigma-Aldrich, St. Louis, MO, USA) from 0.05μg/mL until the cells acquired resistance to 1μg/mL.

### Prediction of differentially expressed-miRNAs (DE-miRNAs)

We conducted RNA sequencing to identify the differentially expressed-miRNAs (DE-miRNAs) in DDP-sensitive SGC7901 and DDP-resistant SGC7901 cell line. Log2 (fold change) (log_2_(FC)) and |-log_10_ (p value) | (|-log_10_(P)|) were chosen as the criteria for identification of significantly DE-miRNAs. Simultaneously, detection of DE-miRNAs was based on log_2_(FC) >1.0 and p-value <0.05. Additionally, all identified DE-miRNAs were then assigned into up-regulated and down-regulated groups, and a heatmap of DE-miRNAs was subsequently depicted through Heml [46].

### Total RNA isolation and qRT-PCR analyses

Human gastric cancer cell lines (SCG7901, AGS, SCG7901/DDP, AGS/DDP) were collected in the logarithmic growth period and treated with 1 mL TRIzol. Chloroform was then added for 15-min at room temperature. Next, isopropyl alcohol was added to the solution and centrifuged to obtain the RNA pellet. Finally, the extracted RNA was dried and stored at -80° C till further experiments. QRT-PCR analyses of miR-95-3p and EMP1 were carried out using the PrimeScript RT reagent Kit and SYBR Prime Script RT-PCR Kits according to manufacturer’s instructions. Transcriptional expression was calculated using the 2^−ΔΔCt^ method [47]. EMP1 expression was calculated relative to GAPDH, while miR-95-3p was calculated relative to U6.

### Lentivirus and shRNA preparation

According to instructions provided by GenePharma, we made Lentivirus constructs that harbored the miR-95-3p inhibitor, its negative control (inhibitor NC), EMP1 overexpression plasmid (EMP1 ov) and empty vector (NC). Furthermore, we acquired specific short hairpin RNAs against EMP1 (sh-EMP1) and short hairpin RNA negative control (sh-NC) from GenePharma Co., Ltd. The sequence of siRNA-339 was: UGACAGCCUGUCAUAUGCCAGUGAAdTdT (sense) and UUCACUGGCAUAUGCAGGCAUGUCAdTdT (antisense). We called the vector p-SUPER (OligoEngine), which harbored the same sequences as p-SUPER-sh-RNA. All constructs were validated by sequencing. Sh-EMP1 and sh-NC were transfected into SGC7901/DDP cell lines in order to knockdown EMP1, while EMP1 ov and NC were transfected into SGC7901/DDP cell lines to upregulate EMP1, based on manufacturer’s protocol. The GC cell lines were treated with miR-95-3p inhibitor to down-regulate miR-95-3p expression.

### Cell transfection

EMP1 shRNA, EMP1 ov, miR-95-3p mimics, or their corresponding negative controls (NCs) were transfected using Lipofectamine® 3000 (Invitrogen), according to manufacturer’s instructions.

### MTT assay and BrdU incorporation assay for cell viability evaluation

Cells (SCG7901, AGS, SCG7901/DDP, AGS/DDP) were seeded onto 96 well plates at a rate of 5000 cells in 100 μL per well. After 48 h, 20 μL of MTT and fresh medium were added, and cultured for an additional 4 h at 37° C and 5% CO2. Finally, cell viability was determined via the OD values that were measured at 570 nm using the enzyme-linked universal micro plate spectrophotometer. In addition, BrdU incorporation assay was also utilized to examine cell proliferation capacity. Next, 10μM BrdU (Thermo Fisher Scientific) was added and cultured for 48 hours, after which colchicine (Sigma-Aldrich, USA) was added to generate a final concentration of 0.1μM. After 48 h, cells were then re-collected and treated using the Giemsa solution (Sigma-Aldrich, USA) for 10 min. Finally, we used a fluorescence microscope to image the stained cells.

### Transwell migration and invasion assay

The 24-well Transwell chamber (8 μm; Corning Incorporated, Corning, NY, USA) were utilized for evaluating invasion capability. Cells were transfected for 48h and then collected. The cell concentration was then adjusted to 10^5^ cells/mL. Next, 100 μL of cell suspension was seeded onto the transwell upper chamber using Matrigel (BD Biosciences, San Jose, CA, USA). Then, 10% FBS was added to all the lower chambers. Next, 24 hours later, the invasive ability of cells was determined through the number of invasive cells that were stained using crystal violet and imaged under a microscope. Simultaneously, the capacity of migration was estimated using the same operation without the matrigel embraced the upper chamber.

### Wound healing test

Cells in the logarithmic growth period were collected and then seeded onto 6-well plates for a 24 hour incubation period. Then, a 200 μl pipette tip was used to develop a wound surface, which was followed by another 24 h of cell culture. The wound area was imaged at 0 and 24 h, respectively. The final wound healing rate was calculated according to the following formula:

(0 h width - 24 h width)/0 h width x 100%.

### Apoptosis study through flow cytometry

Flow cytometry was conducted to study the effect of transfections on apoptosis. The Annexin V-FITC Apoptosis Detection Kit and Cell Cycle Detection Kit were purchased from KeyGEN BioTECH (Nanjing, China). The assay was carried out based on manufacturer’s protocol.

### Dual-luciferase assay

The pGL3 vector (Promega Corporation, Madison, USA) and synthetic EMP1 containing wild-type (WT) or mutated (Mut) region (Sangon, Shanghai, China) were used to construct the reporter plasmids. These plasmids were then co-transfected into cells with miR-95-3p mimics, utilizing Lipofectamine 3000 using manufacturer’s instructions. Additionally, negative control mimics was utilized to generate the control group. After 24 h, Renilla and Firefly luciferase activities were evaluated using the Dual-Luciferase Reporter Assay System (Promega Corp.) and luminometer (Infinite 200 PRO NanoQuant; Tecan Group Ltd., Männedorf, Switzerland).

### Western-blot assay

Cells were collected and lysed using lysis buffer on ice for 30 min. Subsequently, the protein was extracted and the concentration was determined using an ultraviolet spectrophotometer (Thermo Scientific, Waltham, MA, USA). The protein-containing solution was added to the loading buffer and boiled for 5 min to fully denature the proteins. The protein was separated on a SDS-page gel and transferred onto polyvinyl difluoride membranes (Bio-Rad Laboratories, Hercules, CA, USA). The membranes were then incubated with antibodies against MDR1, ABCC1, E-cadherin, N-cadherin, Vimentin, Bax, Bcl2, Bcl-xl, PI3K, p-PI3K (tyr458), Akt, p-AKT (ser473), EMP1 and GAPDH, which was used as internal control. Then observation was performed through the use of an enhanced chemiluminescence reagent (Thermo Scientific, Waltham, MA, USA).

### Animal studies

We set out to elucidate how miR-95-3p promotes DDP-resistance in BALB/c nude mice with gastric cancer. All animals were raised in a pathogen-free environment at Nantong University. The SGC7901/DDP cells were transfected with a miR-95-3p inhibitor and SGC7901/DDP cells were transfected with inhibitor NC. These cells (1×10^6^) were injected into the mammary fat pads of nude mice to construct a GC tumor animal model. When the tumors were palpable, DDP (5 mg/kg) was intraperitoneally injected into the mice every four days. Next, the mice tumor volume was measured every three days. After 21 days of observation, all nude mice were euthanized, and the GC tumor tissues were isolated and photographed. The extracted GC cancer tissues were dyed using HE (hematoxylin-eosin) and also imaged. Proteins within the tissues were measured using IHE (immunohistochemistry) assay.

### Immunohistochemistry

The excised tumor tissue was cut into appropriate size tissue blocks and stored in 4% paraformaldehyde for three days. Next, PBS was used to wash the tissue for 1h, and then ethanol and xylene were used to dehydrate the tissue. The tissue block was immersed into wax solution for 60° C overnight. The tissue block was then embedded, and then cut into 5 μm slices. After placing in a 40° C water bath, the tissue block was dried and stored at 4° C. The tumor tissue sections were heated up to 92-98° C with citrate buffer for 15 min, digested using hydrogen peroxide at room temperature, and then immunohistochemically stained using the corresponding antibodies.

### Statistical analyses

The data is represented as mean plus or minus standard deviation (SD) for triplicate measurements. Statistical significance across different groups was evaluated using the Student’s t test through SPSS (19.0) or Graphpad Prism 7.0. Survival rates were evaluated using the Kaplan-Meier method. A log-rank test was utilized to compare significance. P < 0.05 represents statistical significance.

### Data availability statement

All data generated or analyzed during this study can be requested from the corresponding author.

## Supplementary Material

Supplementary Figures
