# Supplementary material for: MicroRNA-95-3p serves as a contributor to cisplatin resistance in human gastric cancer cells by targeting EMP1/PI3K/AKT signaling
Source: Aging (Albany NY). 2021 Mar 10;13(6):8665–87. doi: 10.18632/aging.202679 (PMC8034895; doi:10.18632/aging.202679)
Supplement: Supplementary Figures [file aging-13-202679-s001.pdf]

## SUPPLEMENTARY FIGURES

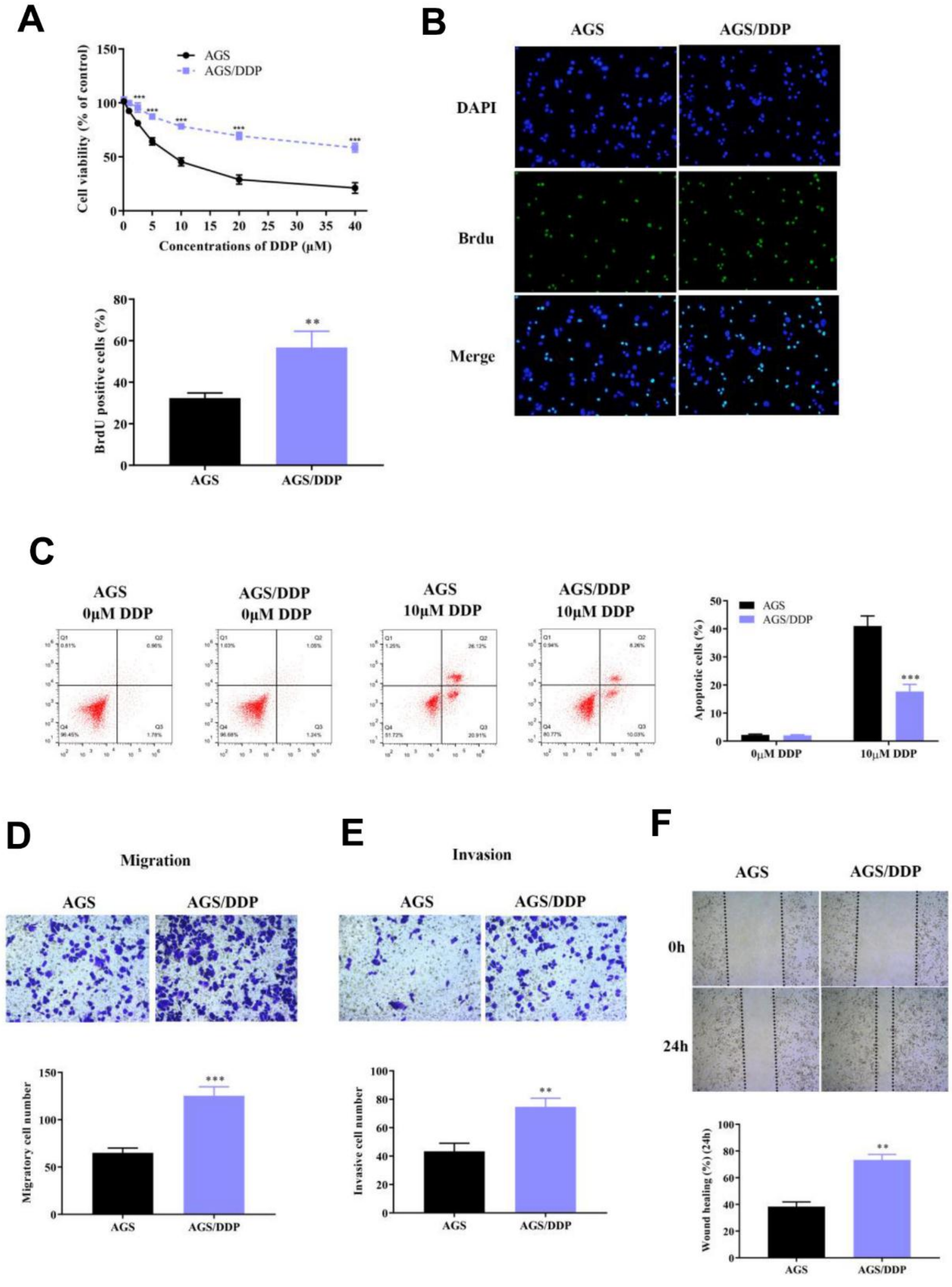


**Supplementary Figure 1. Construction of DDP-resistant GC cell line (AGS/DDP).** (**A**) Cell viability assay demonstrated higher survival rate of AGS/DDP compared with normal AGS cell line when given DDP. (**B**) BrdU assay showed that DDP-resistant AGS cell line manifested significantly higher survival rate than normal AGS. (**C**) Result of flow cytometric assay showed that the apoptosis rate of AGS/DDP was significantly lower than normal AGS. (**D**, **E**) Result of transwell chamber assay indicated more aggressive migrated and invasive capability in AGS/DDP. (**F**) Result of wound healing assay further verified the stronger invasive ability of AGS/DDP. ***p<0.001, **p<0.01 compared with normal SGC7901.

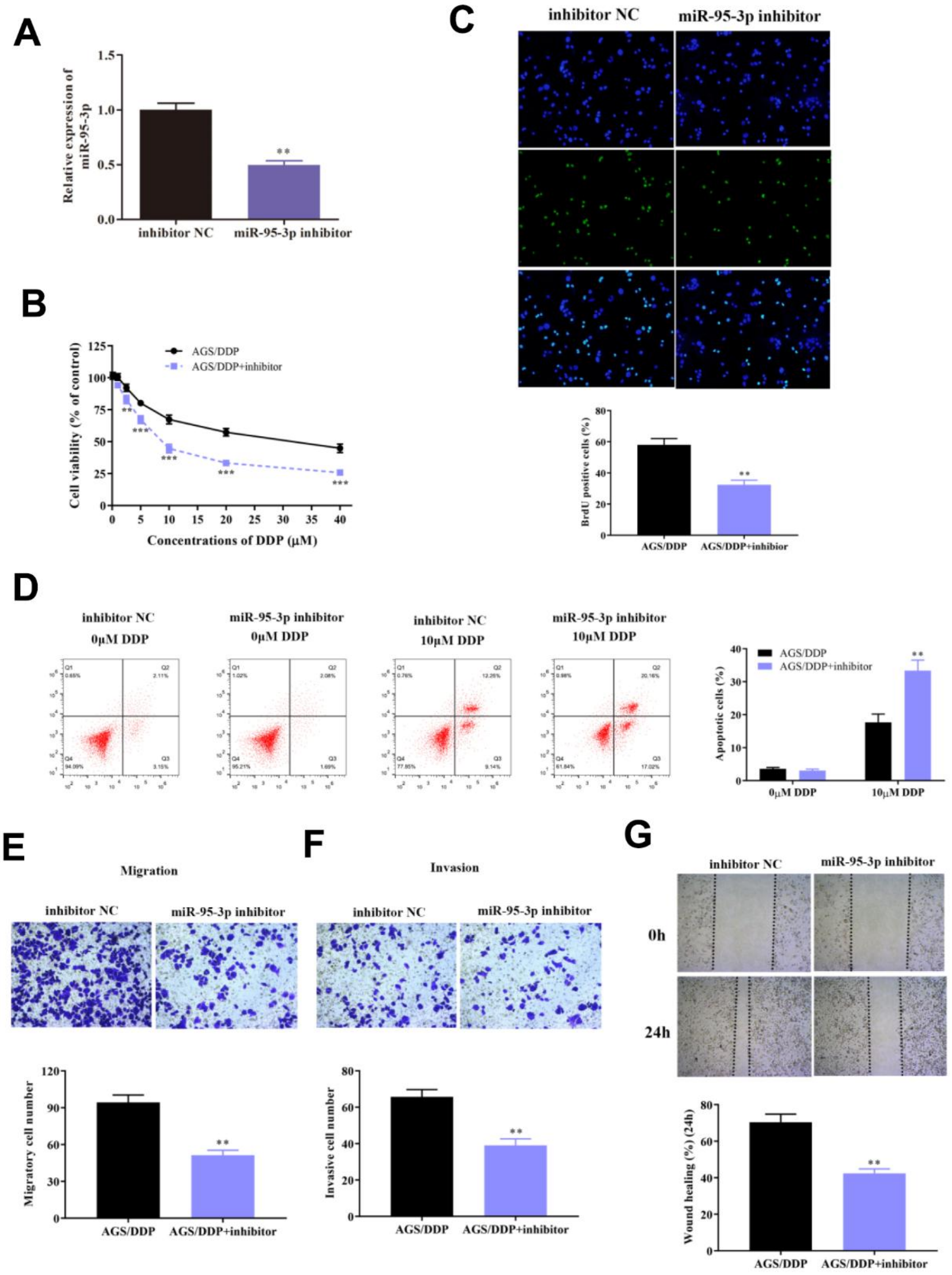


**Supplementary Figure 2. Down-regulation of miR-95-3p resulted in decreased cell viability, weakened invasive and migrated ability, and enhanced cell apoptosis.** (**A**) MiR-95-3p inhibitor was used to decrease the expression level of miR-95-3p. (**B**) MTT assay demonstrated that lower quantities of miR-95-3p induced weaken cell survival rate of DDP-resistant AGS. (**C**) Result of BrdU assay indicated that down-regulation of miR-95-3p lower cell viability of DDP-resistant AGS. (**D**) Cell apoptosis rate of DDP-resistant AGS. (**E**, **F**) Results of transwell assay demonstrated lower cell invasive and metastatic capacity of DDP-resistant AGS when down-regulation of miR-95-3p. (**G**) Wound healing assay indicated that down-regulation of miR-95-3p resulted in decreased invasive ability in DDP-resistant AGS. ***p<0.001, **p<0.01 compared with inhibitor NC.
